# “I did not plan to have a baby. This is the outcome of our work”: a qualitative study exploring unintended pregnancy among female sex workers

**DOI:** 10.1186/s12905-020-01137-9

**Published:** 2020-12-01

**Authors:** Diana Faini, Patricia Munseri, Muhammad Bakari, Eric Sandström, Elisabeth Faxelid, Claudia Hanson

**Affiliations:** 1 Department of Epidemiology and Biostatistics, Muhimbili University of Health and Allied Sciences (MUHAS), 9 United Nations Road, Dar es Salaam, Tanzania; 2 Department of Global Public Health, Karolinska Institutet, Stockholm, Sweden; 3 Department of Internal Medicine, Muhimbili University of Health and Allied Sciences (MUHAS), Dar es Salaam, Tanzania; 4 Ministry of Health, Community Development, Gender, Elderly and Children, Dodoma, Tanzania; 5 Department of Clinical Science and Education, Södersjukhuset, Stockholm, Sweden; 6 Department of Disease Control, London School of Hygiene and Tropical Medicine, London, UK

**Keywords:** Grounded theory analysis: female sex workers, Unintended pregnancy, Contraceptive use, Unmet need for contraceptives

## Abstract

**Background:**

High number of unintended pregnancies—often leading to induced abortions—are reported among female sex workers (FSWs), highlighting a major unmet need for contraception. To better understand barriers to contraceptive use, we explored FSW’s pregnancy perceptions and experiences of unintended pregnancy. We hypothesized that sex work exacerbates barriers to contraceptive use and that FSW’s pregnancy perceptions and experiences of unintended pregnancy influence future commitment to contraceptive use.

**Methods:**

We conducted in-depth interviews with 11 FSWs (January–June 2019) in Dar es Salaam, Tanzania. We purposively sampled FSWs with a positive pregnancy test from those participating in a HIV vaccine preparedness cohort. We used open ended questions to explore how FSWs make decisions when facing barriers to contraceptive use, dealing with unintended pregnancy and adhering to contraceptive use after experiencing unintended pregnancy. All interviews were conducted in *Kiswahili,* audio-recorded, transcribed and translated into English. Grounded theory approach was used to analyse transcripts. Open and selective coding was performed using Nvivo software.

**Results:**

FSWs reported that sex work impedes good contraceptive behaviour because sex workers felt unable to negotiate consistent condom use, avoided health services due to stigma, missed monthly contraceptive supplies because of inconvenient clinic operating hours or skipped contraceptive pills when intoxicated after taking alcohol. FSWs who perceived pregnancy to be a burden terminated the pregnancy because of fear of loss of income during pregnancy or child rearing expenses in case child support was not assured by their partners. FSWs who perceived pregnancy to be a blessing decided to keep the pregnancy because they desired motherhood and hoped that children would bring prosperity. Family planning counselling and availability of contraceptives during postpartum care influenced the initiation of contraception among FSWs. Financial hardships related to childrearing or painful abortion experiences influenced FSWs’ commitment to good contraceptive practices.

**Conclusion:**

Our results demonstrate that FSWs face barriers to initiating and adhering to contraceptive use because of sex work stigma, inability to negotiate condoms and failure to access medical services at their convenience. Our findings underscore the need to integrate contraceptive services with HIV programs serving FSWs in their areas of work.

## Background

Female sex workers (FSWs) have a higher unmet need for contraceptives compared to women in the general population [1, 2]—which is evident from the high rate of unintended pregnancies [3–6], high abortion rates [4, 6–8] and a great desire to prevent future pregnancies [9, 10]. In Tanzania, the incidence of unintended pregnancy among FSWs ranges from 12 to 18 per 100 years of exposure [11, 12], although it is slightly lower than the incidence in other lower and middle income countries of 27 per 100 years of exposure [7]. Although sexual and reproductive health (SRH) services, including contraceptives are an essential component of the comprehensive HIV prevention services for FSWs, there is a notable lack of synergy between provision of HIV prevention and SRH services in most programs [1, 2].

There is evidence that the available SRH services are not tailored to meet the specific needs of FSWs [1, 13]. Stigmatization, discrimination, policing and criminalization of sex work contribute to poor access to contraceptive services [6, 9, 10, 14]. Use of modern non-barrier contraceptive methods among FSWs in Dar es Salaam is 5.7% [3], which is lower than the 40% reported from cities in other sub-Saharan African countries [1, 15–17]. Among users, long-acting revisable contraceptives (Intra-uterine devices and Implants) are less prevalent compared to injectables and oral contraceptive pills [18, 19]. Although both methods are effective, the latter are user-dependent and are prone to incorrect use, discontinuation and frequent switching [5, 17].

Condom failure or condom breakage and non-use among FSWs are common because of physical or sexual violence and drug or alcohol intoxication during sex work [20–23]. Further, sex workers´ financial and emotional dependence to their partners undermine their autonomy to insist on the use of condom or make them forgo condom use for extra payment [4, 18, 24–27], leading to unintended pregnancy [28, 29]. Unintended pregnancies that result in live birth have socio-economic implications that may intensify HIV risk associated with sex work [30–32].

Abortions are a common sought solution among FSWs to reduce the socio-economic repercussions of unintended pregnancy [4, 7, 8, 14]. Induced abortions are illegal in Tanzania [33]. While the penal code in Tanzania authorizes abortion to save a woman’s life, it is silent on its legality to preserve a woman’s physical or mental health [33]. In addition, the law does not allow abortion in cases of rape/sexual violence, which are prevalent among FSWs. This lack of clarity in the law and fear of prosecution pushes women with unintended pregnancies to seek abortions that are often unsafe and which are a major cause of maternal deaths [34, 35].

The high rate of unintended pregnancy and abortion among sex workers are indicative of the limited ability of sex workers to initiate and/or sustain the use of contraceptives. Although a number of quantitative studies have reported factors associated with contraceptive use in this population [3, 10, 19, 36, 37], there is a limited attention to the nuances of how sex work (its daily routine and experiences) intensify these barriers. FSWs in low and middle income countries experience greater risk of repeat unintended pregnancies than women in the general public [7]. However, there is paucity of information on how these repeated experiences of unintended pregnancy and their outcomes (live birth or abortions) influence FSWs’ decision to initiate or adhere to contraceptives. This study aimed to explore how sex work influences contraceptive use and how experiences of unintended pregnancy influence commitment to subsequently use contraceptives. Understanding experiences of unintended pregnancy and contraceptive use among female sex workers is important for informing appropriate interventions to address their SRH needs.

## Methods

### Context of sex work in Tanzania

Sex work, generally defined as exchange of sex for money, goods or other favours [38–40] is diverse and operates in various contexts. In Tanzania, sex work based in entertainment places (bars and/or night clubs) is the most dominant, as well as that operating in lodges and streets. Some FSWs sell sex through formal organized groups (in brothels), others work independently, soliciting clients through mobile phones while others combine sex work with other occupations [41–45].

FSWs may have different types of sexual partnerships including (a) *regular clients*—someone who pays to have sex with a sex-worker on a regular basis, i.e., daily, weekly, monthly (b) *non-regular clients* with whom a sex worker has a single sexual encounter or several sexual encounters over a short period of time, without the expectation of a relationship (c) *husband, boyfriend, or steady partner* who is someone that the FSW regularly has sex with, without receiving any cash payments (d) *transactional partner* with whom sex is exchanged for a favour but not for money, for instance, a partner who offers transportation-a *bodaboda* (motorcycle) driver or a watch-guard who alerts her on police raids.

Sex work, both selling and buying of sex and other sex-work related activities such as facilitating sales, brothel ownership or pimping are illegal and criminal according to the Tanzanian Penal Code [33]. Arrest, police violence, raids and extortion of sex workers are common in Tanzania. Nevertheless, the Tanzanian National HIV Strategic Plan recognizes the need to reach out to sex workers with SRH and HIV services [44].

### The research setting

The study was conducted between January and July 2019 in Dar-es-Salaam, the business capital of Tanzania, which has the highest number of FSWs in the country [46]. The study included informants participating in a vaccine preparedness cohort (PrEPVacc) study, which had recruited HIV negative FSWs aged 18–45 years, living in Dar es Salaam [47]. The main aim of the PrEPVacc cohort study was to determine HIV incidence within a year of follow-up.

### Services offered at the study site

Participants had access to free medical consultation throughout the study. All potential participants were screened for sexually transmitted infections (STI), including Syphilis, as well as Hepatitis B and C. Genital examination for genital ulcers and/or discharge was provided on demand basis. Syndromic diagnosis, drug prescription and/or referral to STI clinic for free treatment was provided. Participants who tested positive for Hepatitis B or C were referred to the Muhimbili National Hospital’s Hepatitis clinic for further management and follow up. HIV testing was performed at enrolment and every three months for enrolled participants. CD4 cell counts and HIV viral load testing were conducted for all HIV infected participants prior to linkage to an HIV clinic of their choice for initiation of antiretroviral therapy.

Study Nurses trained and certified to provide Family Planning (FP) education, delivered FP counselling with a focus on dual methods. Information on barrier methods, short and long acting reversible contraception was provided in groups and in one-to-one sessions during study visits. Male condoms were available onsite and freely provided at each visit. Participants were referred to the FP clinic at Muhimbili National Hospital located within close proximity to the PrEPVacc study site to access pills, injectables, implants, and intrauterine devices. All participants underwent urine testing for pregnancy at enrolment, at 6 and 12-months follow-up visits. Participants who were pregnant were referred to ante-natal clinics of their choice.

### Participants

All informants in the qualitative study were sampled from the 32 women who had a positive urine pregnancy test at the time of enrolment into the PrEPVacc cohort (between October and December 2018). The first author (DF) purposively sampled 18 women by considering demographic and phenomenal considerations to maximize diversity of the informants [48]. Young and older sex workers were selected to reflect variations in their years of experience in sex work as well as their sexual and reproductive health history. Parity and past contraceptive use were phenomenal considerations used in sampling. Participants were invited for interview by a female peer educator who was part of the PrEPVacc study team, and well known in the Dar-es-Salam sex workers’ community.

In-depth interviews were initially conducted with nine of the 18 sampled women who were willing to participate in the study. Three sampled participants could not be reached and six declined to be interviewed (2 were out of town, 1 had a new born baby and 3 reported to be busy). Those agreeing to participate were provided with a daytime interview appointment that was convenient for them. During data collection two additional informants were included in the study making a total of 11 informants. The two informants were considered as “deviant cases” from which, emerging themes could be tested to achieve saturation [49]. One of them was a younger sex worker who had never been pregnant. She provided perspective on contraception and abortions from FSWs with no pregnancy experience. The other deviant case, a HIV positive woman, was included to explore the opinions from preceding informants who had suggested that HIV positive sex workers are less likely to use condoms, (therefore more likely to get pregnant) and that they were more likely to abort the pregnancy in fear of mother-to-child HIV transmission. However, during the subsequent interviews this theme was found not to be consistent and therefore was not further pursued.

Characteristics of the study informants are given in Table 1. At the time of the interviews, five participants were pregnant, three had terminated the pregnancy, two had recently given birth and one had not been pregnant (the additional deviant case). Four women had never used any non-barrier contraceptive. Implants and IUDs were the most commonly used non-barrier contraceptives.

**Table 1:** Characteristics of study participants

Characteristics	Number
Total	11
Age (years)	
< 25	3
25–30	5
30+	3
Education	
Complete primary education	4
Incomplete primary education	2
Secondary/higher education	5
Pregnancy status at time of in-depth interview^a^	
Pregnant	5
Pregnancy terminated	3
Recently given birth	2
Number of previous abortions	
0	3
1	5
2	2
3	1
Number of live children	
0	1
1	6
2	1
3	3
Ever use of hormonal contraceptives	
None	4
Implants/IUDs	4
Pills/injections	3
Context of the unintended pregnancy^a^	
Condom breakage/not using contraceptives	6
Missed contraceptive pill/injection	4
History of using alcohol	
Yes	8
No	3

^a^Excluding one “deviant case”, a sex worker who has never been pregnant

### Design and data collection

We developed the interview guide with topics around sex work, contraceptive use and unintended pregnancy guided by the Health Belief Model [50]. Components of the Health Belief Model were applied to conceptualize how sex workers use a multidimensional approach to make decisions on using contraceptives and when dealing with unintended pregnancy [31, 51]. For instance, the interview guide included probes on how access to contraceptives versus side effects (*perceived barriers*) influence decisions to initiate or continue contraceptive use. The guide also probed on how sex workers perceived their susceptibility to unintended pregnancy given that the decision to use condom was influenced by their partners, and if this *perceived susceptibility* influenced the decision on using dual contraceptives (*perceived benefit*). A pilot interview was conducted by the first author (DF) and transcripts shared with co-authors (CH and EF). Thereafter, the interview tool was revised based on the inputs from the team. During data collection, a meeting was held with researchers from the PrEPVacc cohort, to discuss emerging themes and to make decisions on further questions to be explored in subsequent interviews. In-depth interviews were used for data collection because they are regarded more suitable than focus group discussions for collection of sensitive, personal data [49].

During the interviews, respondents were encouraged to narrate their experiences and reactions upon receiving a positive pregnancy test during enrolment into the PrEPVacc cohort. Participants were asked to describe their thoughts on how pregnancy affected their sex work. The influence of peers, families and partners on unintended pregnancy was also explored. Follow-up questions (probes) were asked in order to give a chance to the informants to explain if the recent pregnancy had influenced their intentions to use contraceptives in the future. Perceptions around induced abortions were explored among both women who had an abortion and those who had not. The interview guide was used in a flexible manner encouraging informants to speak in their own words to allow for new emerging ideas and unexpected information [49]. The interview guide evolved with subsequent interviews accommodating emerging themes based on responses provided by preceding interviews thereby increasing theoretical sensitivity [49]. As a result, later interviews were more focused and covered more concepts albeit risking more narrow and specific responses [49, 52]. For example, the interview with the first informant (In-depth interview conducted in January 2019), covered fewer concepts but provided a long and detailed narration of the issues whereas the interview with the tenth informant (In-depth interview conducted in June 2019) covered more concepts but provided narrower responses.

In-depth interviews were conducted by the first author (DF), in *Kiswahili.* They were conducted in an isolated room at the PrEPVacc study site. The venue was familiar to the informants because they had visited the site for at least three occasions prior to the interview. Informants were also familiar with the research team including DF and PM who were part of the PrEPVacc study team and were involved in enrolment and follow-up interviews of participants in the cohort study. Data collected during enrolment and follow-up interviews included questions on the use and preference of contraceptives. This provided a chance to explore contraceptive use among sex workers other than those already participating in the qualitative interviews. DF and PM took part in delivering pregnancy test results to the women as part of the study visit procedure. This provided a first-hand experience on how women reacted to a positive pregnancy test result because the majority of them had not intended to become pregnant. In all these encounters, personal notes were taken from observations relating to the study question and later used in the analysis process.

### Analysis

All audio-recorded interviews were transcribed and thereafter translated into English by research assistants who were part of the PrEPVacc study. DF read all English transcripts, compared them with the audio files several times, revised any unclear areas, and wrote notes. DF conducted initial line-by-line open coding in accordance with the grounded theory approach [49, 53, 54]. Line-by-line coding allowed many codes to be generated on everything shared by the participants regardless of the relevance to the study questions [49]. Coding was performed using the Nvivo software (Version 12). Codes were developed using keywords that summarized participants’ information. Memos were written to describe the code, and its relevance to the research questions. In some transcripts, text was coded twice or more if the sentences contained more than one relevant aspect.

**Fig. 1:**
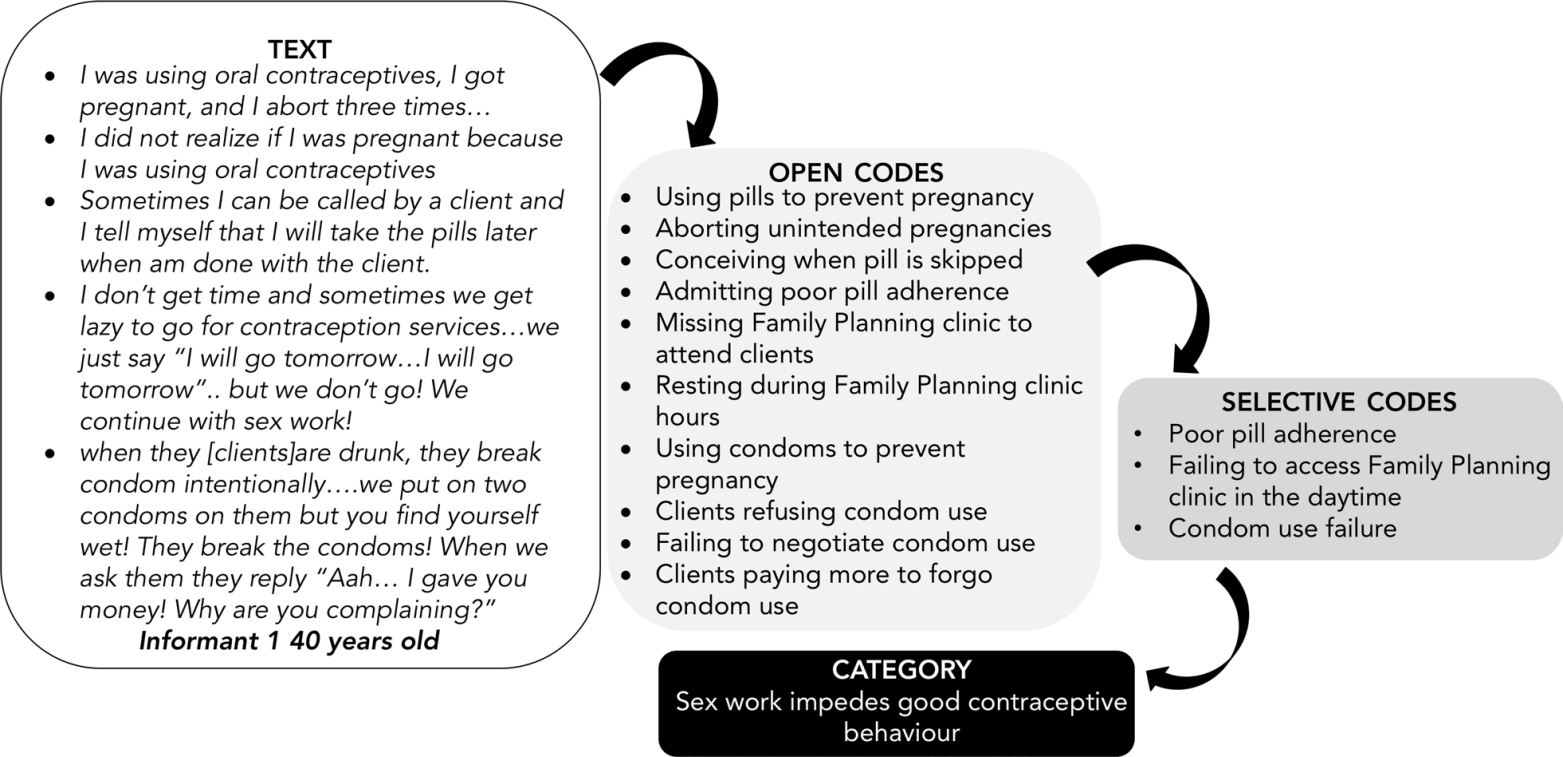
Illustration of grounded theory analysis, moving from text to category

Selective coding was then performed by clustering open codes into categories, going through the transcripts again and deciding which initial codes made the most analytic sense when grouped together [49]. Codes with similar meaning were merged and repetitive codes were deleted. For example, four codes; “a child brings prosperity”, “children bring many blessings”, “children bring luck”, “every child brings with it his/her own blessing” were aggregated to one code “a child is a blessing”. Collating and codifying of the initial codes was done and some codes clustered and a label attached to the category. DF also wrote memos on the categories throughout the analysis to describe how they related to the research question [54]. Transcripts were re-read and in certain instances codes that did not fit in a category were dropped to maintain focus on the research question [49]. An example of moving from text, open codes and selective codes to the category are given in Fig. 1. In the later stage of the analysis, theoretical coding was performed to explain how the codes linked with each other in a particular category and between categories. An overall core-category that captured most important findings of the analysis was selected and linked with the other categories. A model grounded in the data was constructed to illustrate how the codes, the categories, and the core-category related with each other and with respect to the study research question.

## Results

**Fig. 2:**
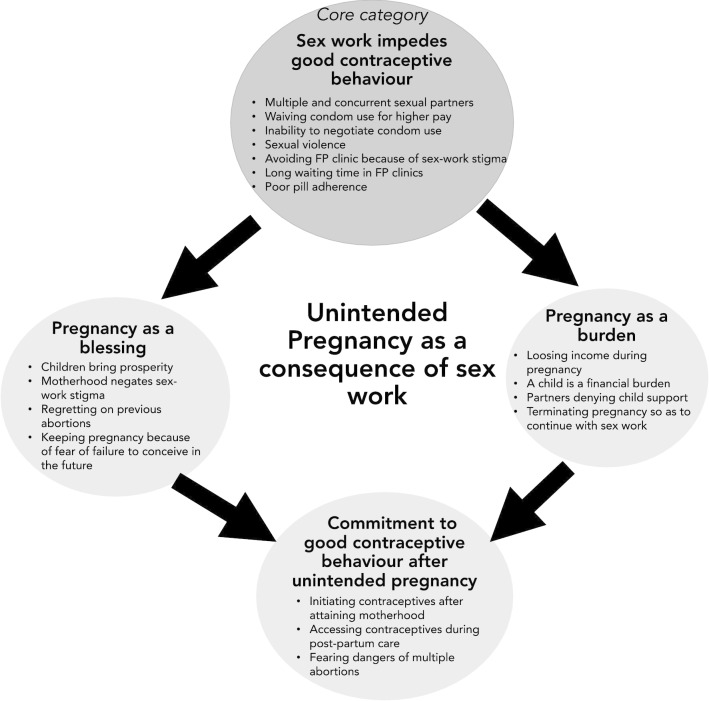
Conceptual categories and core category emerging from grounded theory analysis

This study explored barriers to contraceptive use among female sex workers in Dar es Salaam and examined how their experiences of unintended pregnancies influenced future use of contraceptives. A model illustrating the core category and the main categories emerging from the theoretical coding described earlier was constructed and is illustrated in Fig. 2.

The core category that emerged from the grounded theory analysis was, “Sex work impedes good contraceptive behaviour”, which describes how nuances related to sex work (multiple and/or concurrent partners, inability to negotiate consistent condom use, sexual violence and sex work stigma) create barriers to consistent use of contraceptives. Two other categories, “*Pregnancy as a burden*” and “*Pregnancy as a blessing*”, represent the ambivalent attitudes experienced by FSWs towards unintended pregnancy. The last category, “*Commitment to good contraceptive behaviour after unintended pregnancy*”, describes how experience of an unintended pregnancy, and the effect of abortion or child rearing on the sex worker’s livelihood and wellbeing, influence commitment to good contraceptive behaviour in future.

### Sex work impedes good contraceptive behaviour

Unintended pregnancy was considered an outcome of poor contraceptive behaviour, because sex work impeded good contraceptive behaviour. Sex workers regarded pregnancy as an unavoidable consequence of sex work given the nature of multiple partnerships and inconsistent condom use. Although FSWs were aware of contraceptives and had some knowledge on its effectiveness, they expressed frustration over poor access and adherence to contraceptive use.

#### Sex workers waiving condom use for a higher gain

While condom use was generally desired by the sex workers who wished to protect themselves from not only pregnancy but also sexually transmitted diseases including HIV, they reported that waiving condom use was profitable. Sex workers waived condom use with non-regular clients to get a higher pay. They waived condom use with regular clients/steady partners to maintain good relations for financial and emotional security.

We put on condoms on them and we are very sure of it. But at the end of the act you find yourself wet. They break the condoms. When we ask them, “I put on you two condoms, what happened? “Aah! am not used to it!” or … “I gave you money… why are you complaining?” [Informant 1].

…you may get a man who does not want to use condom and he may even give you extra payment so as to not use a condom! [Informant 2].

For me, if I have a new client, I have to use condoms… if she (any other sex worker) does not use condom, it’s because she is with her regular client … if you are with your regular client you cannot use condom…. so, it is easier to get pregnant from your regular partner than other casual partners! [Informant 9].

#### Sex work stigma from health workers, impedes access to non-barrier contraceptive

Sex workers mitigated inconsistent condom use by opting to use non-barrier contraceptives. However, they reported experiencing stigma from service providers in family planning clinics when seeking contraceptive services. The experience of stigma resulted in avoidance of health services and discouraging peers from seeking similar services.

She (the nurse) started calling her fellow doctor to come and listen to what I was telling her…although I told her alone, in private…. She told all the doctors that I am a sex worker…. I told her, I will buy (the Depo-Provera) so as you can inject me. I told her, if you don’t know how to do it, you could refer me to go to another hospital. So, I decided to leave…. Telling others that I am a sex worker is that not discrimination? I told my friends everything that happened to me there and they were shocked! They said they can’t go there and I told them not to go! [Informant 1].

#### Sex work routine impedes adherence to non-barrier contraceptives

Women explained that contraceptives were readily available in health centres, but long waiting times and inconvenient clinic hours [8 am to 5 pm] made it difficult to seek services. During day-time they are typically sleeping and resting in preparation for working at night. Consequently, they fail to adhere to monthly clinic visits for refills or injections. Pill adherence was also hampered by the tendency to forget taking the tablets when intoxicated after taking alcohol, oversleeping because of working late hours, or skipping the pill when called by a client. They also indicated that the side effects of contraceptives such as nausea, heavy menses interfered with their work which made them opt to risk getting pregnant rather than losing clients/income.

Family planning is there and it is offered for free. They don’t charge you money. You only need to follow their instruction and they will give it to you freely. But the only problem is, when you go there, there is a long line, which I cannot stay/wait because I have other things to do … It takes long to get services. You can be there early morning but because of that long line and a lot of people you end up getting service at 2 pm or 3 pm! [Informant 5].

Yes, I feel sick all the time… I feel sleepy all the time. So, even when a client comes for business, I find it difficult. Yes, their (contraceptive) side effects might make you feel sick after taking them. Because of my job, I didn’t like it (experiencing contraceptive side effects) because I couldn’t make money [Informant 2].

### The value placed on Unintended pregnancy—*a blessing or a burden?*

There were ambivalent attitudes towards pregnancy. Some considered the pregnancy as a blessing, some as a burden and some both as a blessing and a burden. The majority of the women described their pregnancy as “unplanned” or “accidental” but not necessarily “unwanted”. Before pregnancy, their perception of unintended pregnancy—blessing versus a burden—influenced their contraceptive behaviours. The experiences of FSWs during pregnancy and after unintended pregnancy i.e. social or financial implications, greatly influenced the decision of keeping or terminating the pregnancy.

### Pregnancy as a burden—“you must bear and accept the consequences”

Pregnancy was perceived as a burden because of the financial obligations associated with child rearing. Sex workers expressed fears of body changes during pregnancy and the threat of losing income because clients would refuse to have sex with a pregnant sex worker. To lessen the financial burden, sex workers continued working during pregnancy even though they had fewer clients as the pregnancy advanced. Some sought a “potential father” amongst their regular clients, i.e. one who would offer financial support during the pregnancy and after the child was born. Some sex workers decided to terminate the pregnancy if financial support was not assured or if they thought the pregnancy might interfere with their ability to earn.

What will you do? You are pregnant and this is your work and, because you don’t have any other work then you have to continue with sex work… When you are pregnant you are required to rest but because of our work, you have to go on and work. It becomes difficult like when the pregnancy has reached seven or eight months. When it becomes big, with this kind of work, you get some challenges…You can get a man and go with him then at the end he gives you nothing [no payment] [informant 11].

Most of my friends are used to having abortion. They can have sex with whomever and once they get pregnant, they go for an abortion so that they can continue with their sex work. Like at our work place [names the street], when you are pregnant, dah! Men don’t want to be with you! … At the time I was pregnant, I was still doing sex work but not as much as before. I used to go with only two to three customers just to get income for me and for the baby… When you know what you have done you must bear and accept the consequences [Informant 4].

### Pregnancy as a blessing—desiring motherhood

The desire for motherhood was universally expressed by sex workers irrespective of the pregnancy intention. Like other women, sex workers desired children to both care for them and also to have someone to care for. Although participants expressed concerns relating to children-rearing, children were regarded as a blessing. The desire for motherhood was expressed before the occurrence of an unintended pregnancy among sex workers who had never had children and those who had had an abortion. Participants who had a previous abortion expressed regrets. The desire for motherhood was also expressed as a reason why one may not have an abortion in spite of experiencing financial hardships related to pregnancy and child rearing.

There is a saying, every child comes with his/her own blessing… You might take it out [have an abortion] and he/she might be the only one who you have been blessed to have. You might try later (to have children), but not be able to get pregnant. So, it is better if you keep it and see what will the future brings…. [Informant 10].

My friend told me to keep the baby. We have been doing this work for a long time, now it is time to have a baby. It hurts me a lot [the thought of previous abortions]. Sometimes I think a lot and say, if I had older children, they would have helped me in life. They would probably have been doctors…. I wish I would have a baby earlier, maybe she/he will be helping me now…. [Informant 1].

### Committing to good contraceptive behaviour after unintended pregnancy—“My mind was opened”

Having experienced the hurdles of unintended pregnancy, sex workers initiated contraceptive use and committed to adhering to consistent use. Women perceived the experience of an unintended pregnancy as a wake-up call to good contraceptive behaviour irrespective of the pregnancy outcome i.e. having an abortion or live birth. Postpartum initiation of contraception was common among sex workers. Many reported learning about contraceptives and being offered contraceptives after delivery. Women were more likely to accept contraceptive use after delivery due to the myth of infertility associated with contraceptive use at a young age or before childbirth. This misconception was cited as the reason why some sex workers had not used hormonal contraceptives previously, even though they felt that unintended pregnancy was an expected outcome of sex work.

After delivery I then started using contraceptives…. I wasn’t using them before. I was using the calendar method… most of the people that I went to for advice, told me to wait first. They said “You don’t have even a single child and you want to have contraceptives in your body?… use the calendar! Other ways (hormonal contraceptives) are not good!”. If someone does not have a baby yet, contraceptives are going to block the reproductive organs. When I gave birth, people came to give us a seminar. My mind was opened! Right there at the hospital, we were asked if we wanted to get contraceptives…. Some women had injections and others had implants inserted [Informant 2].

Sex workers also expressed painful abortion experience following an unintended pregnancy. They feared having to go through another abortion in the future as it may be fatal or result in infertility. As a result, they expressed a commitment to use and adhere to contraceptives so as to prevent unintended pregnancy.

It’s not that people abort because they don’t know its effects, they know very well but they just look at the immediate outcome. We do not look further to what will happen later. So, you just do it (abort) because it’s important to you at that moment but at the end of the day you can decide to stop and when you want to have a child and you don’t get one. It might be because of the multiple abortions in the past… may be the cervix has experienced some problem like getting loose… or perhaps you were only meant to have one or two children and later you fail to conceive again. You could even die! [Informant 9].

## Discussion

This study explored how sex work hindered good contraceptive behaviour and if experiences of unintended pregnancy influenced future use of contraceptives. Based on the accounts of the FSWs in our study, good contraceptive behaviour was impeded because sex workers [1] were unable to negotiate consistent condom use due to gendered-power inequalities between them and their male clients, (2) avoided health services due to sex work stigma, (2) missed monthly contraceptive supplies in family planning clinics because of inconvenient clinic operating hours or (4) skipped pills when intoxicated after taking alcohol. We also found that although sex workers desired *motherhood*, there was a great desire to use contraceptives in order to avoid unintended pregnancy and its associated health, social and economic burden. Experiences of unintended pregnancy and the associated hardships of losing income and rearing children led to future commitment to initiate and/or adhere to contraceptive use.

Desire for motherhood was universally expressed by sex workers as important even though unintended pregnancy was perceived as an adverse outcome of sex work. Unintended pregnancy has previously been described as a burden by FSWs as it results in losing clients and income, having to spend money on an abortion and risking post-abortion complications [4, 55]. In order to balance the consequences of their decisions e.g. aborting, paternity, working during pregnancy and after delivery, studies show that sex workers make pragmatic, complex and multiple decisions that cater for not only the immediacy nature of the unintended pregnancy but also in anticipation for a better future [27, 31, 55]. Nevertheless, sex workers in this study, as those in other settings, desire to have children [27, 56, 57].This is because motherhood is regarded as empowering as it symbolizes womanhood, childrearing and marriage, attributes that earn women a respectable and accepted role in the society [58, 59]. Motherhood earns sex workers respect amongst peers and family [30, 59–61] and therefore counter the stigma associated with their role as sex workers [27, 30, 55, 62]. This high value placed on motherhood by sex workers may undermine sex workers´ agency to use contraceptives, especially before having any child and if she perceives children to be a blessing and a source of her future economic support.

Sex-workers’ access to non-barrier contraceptives was impeded by inconvenient family planning clinic hours, sex work stigma and shame in medical facilities. The reluctance of health care workers to provide care to FSWs and avoidance of health services by FSWs is prevalent in sub–Saharan Africa [9, 14, 63, 64]. A qualitative study in Kenya identified long waiting time, user fees and inconvenient operating hours as factors that impede contraceptive access [65] whereas in Cameroon, experiences of health-care related stigma was common among FSWs using non-barrier contraception [6]*.* To reduce stigma and discrimination against sex workers in clinical settings, health care workers should be engaged in sensitization meetings to raise awareness of FSWs’ needs and the hardship they face, in order to reduce vulnerability when seeking care. Such meetings should involve FSWs so as to challenge popularly held stigmatized notion of sex work and highlight how health care workers could change their attitudes towards FSWs. Collectively, FSWs should be empowered to discuss health care workers’ actions they consider improper. Such structural interventions addressing stigma against FSWs by engaging and involving the FSWs community are widely advocated by the World Health Organization [66, 67] and have been shown to be effective in South Africa and India [14, 68–71].

We found that use of dual contraceptives (condoms and a non-barrier contraceptive method) among FSWs in the study was not universal even though the women acknowledged repeated experiences of unintended pregnancies and abortions. In Tanzania, only 29% and 16% of FSWs consistently use condoms with non-regular and regular sexual partners, respectively [3, 41, 72]. Given the risks of sex work i.e. intimate partner violence, multiple and concurrent partnership, relying on condoms alone for preventing both HIV and pregnancy among FSWs is implausible. Inability of sex workers to negotiate condom use with partners has been widely reported in literature and is largely linked to gendered-power inequalities [4, 21] with sex workers waiving condom use for fear of losing emotional and economic support [21, 73–76]. The lack of autonomy to insist on consistent condom use underscores the need for dual contraception among FSWs, particularly long acting reversible contraceptives. Similar to findings in other studies [3, 4, 10, 15], FSWs in this study reported using injectables or oral contraceptive pills when they got pregnant. These contraceptive methods are user dependent and are prone to inconsistent use and discontinuation because of poor daily pill adherence, failure to attend clinics for monthly supplies [4, 17] as well as menstrual side effects [19, 36]. Forgetting to take pills is a common reason for contraceptive use failure, with some sex workers reporting taking pills only on the day they have sex [4].

Sex workers need more specifically targeted services. Experience from a community-based study among FSWs in Tanzania that included community-led drop-in centre and mobilization activities as well as venue-based peer education, condom distribution, and HIV testing, showed significant reduction in HIV incidence [77]. Expanding such community-based model which reaches out to FSWs in their areas of work, to further include family planning services can be an effective strategy to reduce unintended pregnancy among FSWs. There is a growing body of research suggesting that integration of HIV and family planning services in a single location as well as providing multiple contraceptive options suited for fertility intentions increase demand and uptake of contraceptives [18, 78–80]. One study in Uganda showed that FSWs were more likely to receive contraceptive services if they had accessed HIV testing [10]. Linking FSWs to SRH and HIV services is vital for navigating barriers to contraceptive access. Although drop-in centres are preferred by sex workers [14], they have low coverage. Additionally, their sustainability is uncertain as they are mostly run by Non-governmental organizations or research groups and are therefore largely disconnected from the public facilities where majority of sex workers seek care. A more systematic approach is already being used by the Tanzania’s Ministry responsible for health through engaging sex workers in dialogue pertaining to provision of HIV pre-exposure prophylaxis in health facilities. Such platforms provide avenues for advocating for implementation of non-stigmatizing and non-discriminatory comprehensive SRH services in public facilities as stipulated in the country’s national guidelines [44].

In this study, postpartum initiation of non-barrier contraceptives was common following an unintended pregnancy. This could be because information and contraceptives are universally provided to women in postpartum clinics, hence sex workers are less likely to encounter stigma. The acceptance of initiating dual contraception following unintended pregnancy was also reported by a study among FSWs in Uganda [10]. Another study among FSWs in Kenya similarly reported initiation of long-acting reversible contraceptives after experiencing unintended pregnancy [19]. Thus ensuring provision of family planning counselling and methods in postpartum and in post abortion–care services may be a key intervention area for FSWs.

This study has limitations. First, generalization of the findings to other contexts may be limited because of the specific urban setting as well as the fact that the qualitative study was conducted within a vaccine preparedness cohort study. The parent cohort included FSWs with specific inclusion criteria who were recruited by a chain-referral mechanism by members in their social network. It is likely that, FSWs in this study were part of a larger network, had better health-seeking behaviour including better contraceptive practices compared to those in their wider community. In addition, FSWs enrolled in the cohort were followed-up at three-month intervals and received counselling on risk reduction and use of contraceptives. Second, the in-depth interviews were conducted on average four months after the positive pregnancy test recorded during enrolment into the PrEPVacc cohort. Consequently, by the time participants were interviewed, some had terminated their pregnancy, some were still pregnant (one woman was at term) and some had already given birth. Although this diversity allowed us to explore how experiences and decision around unintended pregnancy differed which ensured that themes developed were comprehensive, there is also a risk that this extensive period of data collection may have resulted in inconsistency, meaning that interview questions may not have covered same areas for all participants [52].

In spite of the above limitations, there are several strengths to this study. Unlike quantitative studies, which are often restricted to close-ended questions on contraceptive use, we explored the specific context surrounding each unintended pregnancy in relation to previous use and/or adherence to contraceptives, as well as a reflection of future commitment to contraceptive use. To improve credibility of the findings, the first author had prolonged engagement with the participants before commencement of data collection e.g. during the scheduled interviews as part of the parent cohort study procedure and during PrEPVacc participant engagement meetings that were held weekly. The study team included local and international researchers from different professions and cultures. They provided inputs and critical comments in the conceptualization of the study, design of the interview guide, review of the analysis and emerging themes. The diversity in the research team allowed the first author to asses her role in the research process during the interviews and interpretation of the findings. During the analysis, the first author shared preliminary results in meetings with other qualitative researchers. These meetings included negotiating interpretation of the results and selection of the core-category derived through grounded theory analysis. This was also done as part of the trustworthiness assessment of the qualitative research in order to ensure that analysis and presentation of the emerging themes were independent of the subjectivity of the first author who conducted the interviews.

## Conclusions

Our results indicate that initiation and adherence to contraceptive use among sex workers is impeded by a number of contextual factors such as gender-negational power, poverty as well as sex work stigma. Although some of these contextual factors are not unique to sex workers, sex work by its nature exacerbates them, and hence makes it difficult to achieve good contraceptive behaviour, including consistent use of dual-methods. Our results indicate that the health needs among FSWs extend beyond HIV and highlight the importance of removing structural barriers for optimizing coverage of health services. Further, our results underscore the need to integrate SRH services with HIV programs serving FSWs in their work areas, including contraceptive counselling and provision of a wide range of non-barrier contraceptives—particularly, long-acting reversible contraceptives—that meet the fertility desires of these women. De-stigmatization of sex work is also crucial for the achievement of such programs.

## Data Availability

The datasets used and analysed in the study are available from the corresponding author on reasonable request.

## Abbreviations

FP: Family planning
FSWs: Female sex workers
HIV: Human immunodeficiency virus
IUDs: Intra-uterine devices
MUHAS: Muhimbili University of Health and Allied Sciences
SRH: Sexual reproductive health
STI: Sexually transmitted infections
